# Natural depolymerization of waste poly(ethylene terephthalate) by neutral hydrolysis in marine water

**DOI:** 10.1038/s41598-021-83659-2

**Published:** 2021-02-24

**Authors:** Dorin Stanica-Ezeanu, Danuta Matei

**Affiliations:** Petroleum-Gas University of Ploiesti, Ploiești, Romania

**Keywords:** Environmental sciences, Engineering, Materials science

## Abstract

Polyethylene terephthalate (PET) is one of the most widely used materials for food packaging and fishing nets. After use it become waste and, due to poor collection, most will be found floating in marine waters. This paper presents the results of a study of PET depolymerization by hydrolysis. We observed that marine water is a perfect reactant because it contains a multitude of metal ions that act as catalysts. A first-order kinetic model was developed and experimental data fitted to it. An activation energy of 73.5 kJ/mole and a pre-exponential factor of 5.33 × 10^7^ h^–1^ were obtained. Considering that the global ocean is a huge batch reactor operating under isothermal conditions, the solution of the mathematical model shows that in tropical regions only 72 years is needed for total and only 4.5 years for 50% PET conversion.

## Introduction

Global food consumption increases day by day and, in the same manner, greater amounts of plastic packaging are released in the environment with a massive impact on the living world, especially on marine life. The impact of waste plastic on the marine environment, taking into consideration the effects on marine life and the financial losses in tourism and the fishing industry represents over $13 billion per year^1^.

Industrial development has produced an exponential increase in plastic production reaching 311 million metric tons (MT) in 2014, and the forecast is that this will be doubled by 2035 and almost increased fourfold by 2050^2^. The conclusion is that in the marine environment there will be more waste plastic than fish, and many studies have shown that this plastic debris seriously affects marine life^3–7^.

According to Jambeck et al., in 2010 a total of 275 MT of plastic waste was generated by 192 coastal countries, and an estimated 4.8–12.7 MT was discharged into the marine environment. They observed that population size and the efficiency of waste management systems in these coastal countries are the determinant factors of the uncollected mass of waste plastic becoming plastic marine litter. If the waste management infrastructure is not improved, the forecasts are bleak: in 2025 the plastic waste discharged into the global ocean by the people of coastal countries will have increased by an order of magnitude^3^.

Even in the absence of accurate data, most studies claim that from 5 to 50% of total plastic waste discharged into marine waters is polyethylene terephthalate (PET), a thermoplastic widely used in the manufacture of fishing nets, soft-drink bottles and photographic film^8,9^.

Since PET does not decompose readily in nature, it may be chemically decomposed to yield the original feedstock monomers, terephthalic acid (TPA) and ethylene glycol (EG). One of the most interesting chemical processes for PET depolymerization is hydrolysis. This process, in the laboratory or in industrial plants, occurs at high temperature and pressure under acid, base or neutral catalysis^10^.

Neutral hydrolysis is carried out with water or steam using water-soluble salts as catalysts. Paszun and Spychaj^11^ reported a great number of patents and papers that describe different methods of PET degradation by hydrolysis using alkali-metal acetates as effective catalysts. Campanelli et al.^12^ studied the effect of zinc catalysts and observed a higher conversion when the reaction was carried out at temperatures higher than 250 °C, at which PET is in the molten state. Also, Güclü et al.^13^ used zinc acetate and KOH for depolymerization of waste PET by hydrolysis and they observed that the presence of xylene will increase the depolimerization degree at lower temperature, pressure and amount of water. Despite the fact that hydrolysis is an ecologically sound way to transform waste PET into valuable products, the process is not applied on the industrial scale because of the high energy consumption and the low purity of the final products. Michalski^14^ offered the solution of TPA purification by crystallization from solutions of TPA dissolved in caprolactam or NaOH solution.

In this paper we present the results of a study of the hydrolysis of waste PET using novel catalysts such as NaCl, CaCl_2_, NaHCO_3_, KHCO_3_ and even marine water, compared to the widely used acetate catalysts (Zn, Cu, Co, Cd) and MnSO_4_. This study shows that in the marine environment the depolymerization of waste PET by hydrolysis is a natural process with a rate of reaction determined only by the surface water temperature.

## Experimental

### Materials

PET chips (3 mm × 5 mm) were obtained by cutting up a PET beverage bottle. The average molecular weight was 24 800 g/mol. The chips were washed in hot water (80 °C) and dried for 2 h at 105 °C in an oven. The chemicals used as catalysts are: NaCl, CaCl_2_, NaHCO_3,_ KHCO_3,_ (CH_3_COO)_2_Zn, (CH_3_COO)_2_Cu, (CH_3_COO)_2_Co, (CH_3_COO)_2_Cd, and MnSO_4_. All reagents were of analytical grade and used without further purification. For our study we used two samples of marine water: the first was from the Black Sea (Cape Midia, Romania) and the second from the Atlantic Ocean (Costa Caparica, Portugal). The contents of major ions present in these two samples of marine water are presented in Table 1.

**Table 1:** Major ion composition of marine water.

Ion type	Black Sea	Atlantic Ocean
Chloride (Cl^−^), ppm	8850	19,465
Sodium (Na^+^), ppm	4930	10,790
Sulfate (SO_4_^2−^), ppm	1350	2695
Magnesium (Mg^2+^), ppm	595	1305
Calcium (Ca^2+^), ppm	194	410
Potassium (K^+^), ppm	174	390
Bicarbonate (HCO_3_^−^), ppm	70	145
Bromide (Br^−^), ppm	n.a.	65
Salinity, (g/kg)	16.15	35.5

### Analysis

A Netzsch TG 209 F3 microbalance was used for thermogravimetric analysis (TGA) of the solid product obtained in the PET hydrolysis reactor in order to determine the TPA content in the sample. Approximately 5 g of sample was heated with a rate of 10 °C/min in an aluminum crucible under a nitrogen atmosphere and the mass loss of the sample recorded. The recorded diagram of the sample was compared with that of pure TPA obtained under the same operating conditions.

### Hydrolysis reaction

Ten grams of waste PET (flakes of 3 mm × 5 mm) and a solution of 2 g of catalyst in 100 mL of water were placed into a stainless-steel batch reactor equipped with a magnetic stirrer and a temperature control system. A purge valve was used to fill the reactor with nitrogen in order to replace the air and to increase the internal pressure to over 3 MPa in order to keep the water in a liquid state at the reaction temperature (190–215 °C). The reactor was heated with an electric furnace and attaining the reaction temperature was defined as the start of the reaction. After the required reaction time the autoclave was removed from the electric heater and cooled rapidly to room temperature. Finally, the obtained solid product in the reactor was separated by filtration from water, catalyst and the EG obtained as byproduct.

The TPA on the glass filter was washed with warm water and dried at 105 °C for 2 h in an oven. The dried solid product was weighed and analyzed to calculate the PET conversion and the yield of TPA. In the case of bicarbonates as catalysts, the final product of PET hydrolysis was sodium terephthalate, which remained in solution; the TPA was precipitated by the addition of 2 N H_2_SO_4_ followed by washing, drying and weighing. When sea water was used as reactant, the catalyst was a mixture of salts (chlorides, bicarbonates, sulfates, and bromides) dissolved in this water, and the reaction product was a mixture of TPA (solid) and sodium terephthalate (dissolved in water). To recover the TPA from this mixture we started with a filtration step in order to separate the precipitate already formed in the reactor, followed by a second step of treating the liquid separated in the first step with a solution 2 N H_2_SO_4_ to precipitate the TPA from the sodium terephthalate solution. Finally, the TPA recovered by filtration from both steps was washed, dried and weighed.

The PET conversion and TPA yield were calculated by following equations:

$$ {\text{PET conversion }} = \, \left\{ {\left( {{\text{W}}_{{{\text{PET}},0}} {-}{\text{ W}}_{{{\text{PET}},{\text{f}}}} } \right)/{\text{W}}_{{{\text{PET}}}} ,_{0} } \right\} \times {1}00, $$

$$ {\text{TPA yield }} = \, \left\{ {{\text{W}}_{{{\text{TPA}}}} /\left( {{\text{W}}_{{{\text{PET}},0}} {-} {\text{W}}_{{{\text{PET}},{\text{f}}}} } \right)} \right\} \times {1}00. $$

where W_PET,0_ = the initial weight of PET (g); W_PET,f_ = the residual weight of PET (g); and W_TPA_ = weight of TPA obtained by hydrolysis (g).

## Results and discussion

### Hydrolysis of PET using a single catalyst

Hydrolysis of PET is a chemical reaction carried out in the presence of a catalyst, usually a metallic salt soluble in water and the mechanism shows that the metal ion attacks the C=O bond producing an electrolytic destabilization in the neighboring bonds, followed by cleavage of the polymer chain^12^.

Our experiments show that well known catalysts, such as metal acetates (zinc, cobalt, copper, cadmium etc.) or MnSO_4_, can be replaced by NaCl, CaCl_2_, NaHCO_3_ or KHCO_3_ with the same or better results under the same reaction conditions. The only difference is that, in the case of carbonic salts, the final product is sodium terephthalate, which being soluble in water will remain in the liquid phase. To separate the TPA from the liquid mixture, it is necessary to precipitate it by a treatment with a stronger acid, such as H_2_SO_4_.

The results of the experimental study are presented in Table 2.

**Table 2:** Experimental data of PET depolymerization by hydrolysis using a single catalyst.

Catalyst/PET Weight ratio g/g	H_2_O/PET ratio g/g	Reaction time, min	Reactor temperature, °C	Reactor pressure, MPa	PET conversion, %	TPA yield, %
**Catalyst: 2 g (CH**_**3**_**COO)**_**2**_** Zn**						
0.7	14	135	215	4.0	73.5	81.5
**Catalyst: 2 g (CH**_**3**_**COO)**_**2**_**Cu**						
0.1	20	120	215	4.0	87	79.5
**Catalyst: 2 g (CH**_**3**_**COO)**_**2**_**Co**						
0.1	20	165	217	4.0	86	76.5
**Catalyst: 2 g (CH**_**3**_**COO)**_**2**_**Cd**						
0.1	20	210	214.5	4.0	85.4	80
**Catalyst: 2 g MnSO**_**4**_						
0.1	20	120	213	4.0	85	84
**Catalyst: 2 g NaCl**						
0.2	10	180	196	3.1	82	80
**Catalyst: 2 g NaHCO**_**3**_						
0.1	10	150	196	3.0	80.75	95
**Catalyst: 2 g CaCl**_**2**_						
0.1	5	180	198	3.95	65	88.5
**Catalyst: 2 g KHCO**_**3**_						
0.1	5	180	190	3.85	60	98

### Hydrolysis of PET using a double catalyst

The experimental study showed that inorganic salts such as NaCl, CaCl_2_ or bicarbonates of sodium and potassium are very good catalysts for the hydrolysis of PET; thus, we tried to use a combination of two salts (NaCl + CaCl_2_ and NHCO_3_ + KHCO_3_) dissolved in water; the results are presented in Table 3.

**Table 3:** Experimental data of PET depolymerization by hydrolysis using a double catalyst.

Catalyst/PET Weight ratio g/g	H_2_O/PET ratio g/g	Reaction time, min	Reactor temperature, °C	Reactor pressure, MPa	PET conversion, %	TPA yield, %
**Catalyst: 1 g NaCl + 1 g CaCl**_**2**_						
0.4	20	120	197.5	3.3	77.5	90.0
**Catalyst: 1 g NaHCO**_**3**_** + 1 KHCO**_**3**_						
0.4	20	120	195	3.5	86.5	95.7

This combination of two catalysts show the same efficiency as single catalysts widely used in other experimental studies^10–14^ and, was the engine of the researches using marine water as reactant and catalyst also.

### Hydrolysis of PET with marine water

The marine waters contained a mixture of metallic ions (Na^+^, Mg^2+^, Ca^2+^, K^+^) which act as a multitude of catalysts to produce multiple electronic destabilization of the ester bonds in the polymeric chain. The result is a random depolymerization of PET, decreasing the chain length and finally obtaining the monomers TPA and EG.

The experimental results are presented in Table 4.

**Table 4:** Experimental data of PET depolymerization by hydrolysis with marine water.

Catalyst/PET weight ratio g/g	H_2_O/PET ratio g/g	Reaction time, min	Reactor temperature, °C	Reactor pressure, MPa	PET conversion, %	TPA yield, %
**Catalyst: 100 g Black Sea water (salinity: 16.15 g/kg)**						
0.16	10	120	195	3.0	86	92.5
**Catalyst: 100 g Atlantic Ocean water (salinity: 35.5 g/kg)**						
0.35	10	120	205	3.3	87.5	96.0

**Figure 1:**
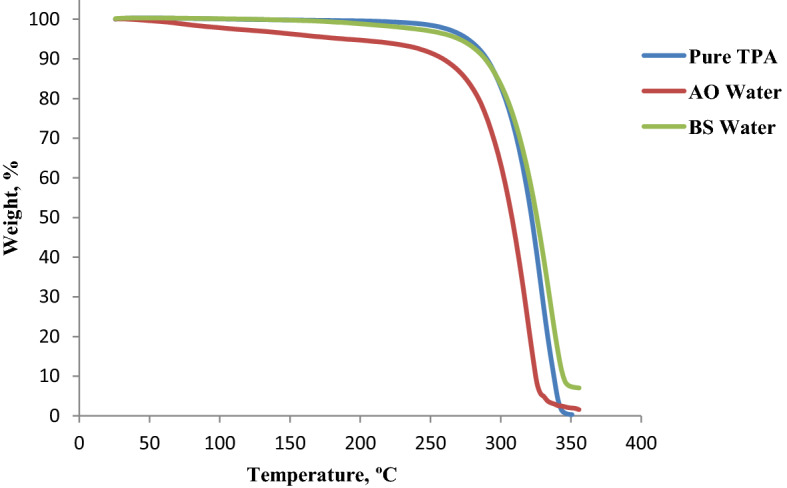
TGA diagrams for AO and BS water vs. pure TPA.

Thermogravimetric analysis (TGA) of the solid product obtained by PET hydrolysis with marine water shows that TPA is the main compound in this product. In Fig. 1, the TGA diagrams for Black Sea (BS) and Atlantic Ocean (AO) waters are compared with that of pure TPA to demonstrate the high content of TPA but also the presence of other compounds (oligomers) of PET.

### Kinetic study of PET hydrolysis in marine water

All experimental data show that marine water could be a very effective catalyst for the hydrolysis of PET waste, and we used the experimental data to calculate the kinetic parameters of the reaction. Taking into consideration that one of the reactants (H_2_O) is in excess over the other (PET), the reaction is pseudo-first-order and experimental data were fitted with activation energy Ea = 73.5 kJ/mol and pre-exponential factor A = 5.33 × 10^7^ h^–1^.

### Natural hydrolysis of waste PET in marine water

The presence of waste PET floating in marine water is an opportunity to start a natural hydrolysis process with a rate of reaction determined by local operating parameters (Supplementary Material).

Using these kinetic data, we can calculate the time of reaction by natural hydrolysis for existing PET waste in marine waters. It is easy to imagine that the global ocean is a huge batch chemical reactor where these two reactants (PET and H_2_O) interact with the participation of metal ions as catalysts. Because of the excess of water, the reaction is first-order and the process can be considered isothermal because the temperature of the marine water is almost constant for a long period of time. From the material balance of the batch chemical reactors operated in isothermal conditions, in the case of a first-order chemical reaction, the time of reaction (t_r_) for a fixed PET conversion is calculated by the following equation^15^:

1 $${{t}_{r}}_{= \frac{1}{k}\cdot ln\frac{1}{1-{X}_{PET}}}$$

where: k = reaction rate constant (Arrhenius), h^−1^, X_PET_ = PET conversion, molar fraction.

The results obtained for different marine waters of different temperatures are presented in Table 5.

**Table 5:** Waste PET depolymerization time by natural hydrolysis in marine environment.

Global Ocean area	Surface average temperature^16^, °C	Waste PET depolymerization time (years) for a conversion of	
		50%	99.999%
**Atlantic/Pacific Ocean**			
Arctic zone	0.5	162	2692
Temperate zone	17	26	428
Tropical zone	30	7	116
**Indian Ocean**			
Southern zone	3	121	2008
Tropical zone	35	4.5	72
**Mediterranean Sea (Malta)**			
Cold season	10	55	910
Warm season	27	9	155

These results show that depolymerization of PET in marine water by natural hydrolysis is strongly influenced by the surface water temperature. When the temperature increases the total depolymerization of PET is achieved in a shorter period of time; for instance, 72 years at 35 °C. The hydrolysis of PET proceeds day and night at any temperature, even in the Arctic zone where 50% of PET is transformed into monomers and oligomers in 162 years.

**Figure 2:**
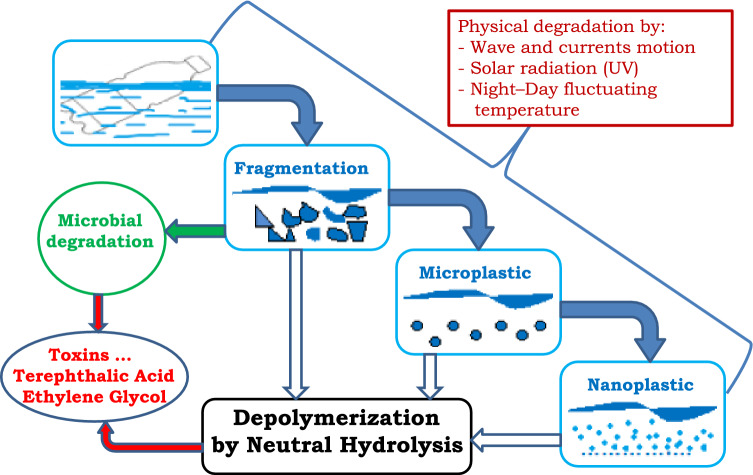
Factors involved in PET waste depolymerization in marine environment.

By these calculations, we demonstrated that the forecasts regarding the total degradation of waste PET by natural processes, of hundreds of years, even 450 years^17,18^, are wrong. Besides this chemical process, the process of depolymerization can be shortened if biological depolymerization^19–22^ and physical destruction of the polymer by mechanical forces and sunlight photodegradation^23,24^ are taken into consideration. All these natural factors involved in the degradation and depolymerization of PET waste floating in marine water are presented in Fig. 2.

At the moment, we have insufficient data to evaluate the influences of physical and biological factors, but by reduction in the size of floating fragments the overall time of depolymerization could be reduced by 10–20%. The smaller pieces are transformed more rapidly into TPA and EG monomers. These are not environmentally friendly, especially the acid, which will decrease the pH of marine water with side effects on local fauna and flora.

## Conclusions

Our study shows that PET depolymerization by neutral hydrolysis using marine water is a continuous process that transforms the long polyester chains into TGA and EG. The global ocean acts as a huge batch reactor where waste PET hydrolysis proceeds in isothermal conditions following first-order kinetics. Using the mathematical model of an isothermal batch reactor, we calculated the time of reaction for 50% and 99.999% (total) PET conversion, which show that the time of PET depolymerization does not depend of the dissolved salt concentration; temperature is the only factor that controls the rate of depolymerization. Thus, anywhere on the globe the time of reaction is the same at the same temperature. According to our research, the time of reaction for a PET conversion of 50% at 35 °C is only 4.5 years in any tropical zone of the Atlantic, Pacific and Indian Oceans or the Caribbean Sea. Also, total PET depolymerization, at a temperature of 30 °C needs only 162 years in any marine water on the globe. All these calculated data provide precise information about the period of depolymerization of waste PET floating in marine waters and correct old estimations of more than 400 years for the total degradation of waste PET.

However, our calculated times, which take into consideration only the chemical depolymerization by hydrolysis, could be reduced by 10–20% if, simultaneously, microbial degradation and physical processes are added as effective factors in the degradation of solid PET waste floating in marine waters. It is undeniable that smaller pieces are transformed more rapidly into the monomers TPA and EG, and the overall disappearance of the solid waste will be accelerated.

Finally, all these data show that nature is fighting human aggression with all its weapons (chemical, physical, and biological) in order to maintain a clean environment for future generations.

## Supplementary Information

Supplementary Information.
