# Supplementary material for: Natural depolymerization of waste poly(ethylene terephthalate) by neutral hydrolysis in marine water
Source: Sci Rep. 2021 Feb 24;11:4431. doi: 10.1038/s41598-021-83659-2 (PMC7904861; doi:10.1038/s41598-021-83659-2)
Supplement: Supplementary file 1 — Supplementary Information. [file 41598_2021_83659_MOESM1_ESM.pdf]

Supplementary information

To the paper: **Natural depolymerization of waste Poly(ethylene terephthalate) by neutral hydrolysis in marine water**

Authors: Dorin Stanica-Ezeanu. Danuta Matei

Petroleum-Gas University of Ploiesti, Romania

Kinetic study for PET depolymerization by hydrolysis using Black Sea water

The experiments were carried out in a Batch reactor with excess of water and in isothermal conditions. The results are presented in the following table:

Temperature, °C	Time of reaction, t_r h	Conversion X_{PET} %wt	Reaction rate constant k, h^{-1}	ln k h^{-1}	1/T, K^{-1}
150	2	7.8	0.0447	-3.11	0.0236
167	2	18.2	0.1	-2.3	0.0227
185	2	35.5	0.219	-1.51	0.0218
200	2	55.7	0.407	-0.9	0.0211

Because of excess of water the order of PET depolymerization reaction by hydrolysis is pseudo-one and the rate of reaction is a function of PET concentration only:

$$(-r_{PET}) = k \cdot C_{PET} \qquad (1)$$

From mathematical model of the Batch reactor operated at constant volume (liquid phase) and in isothermal conditions, the reaction time is calculated as:

$$t_r = \frac{1}{k} \cdot ln \frac{1}{1 - X_{PET}} \qquad (2)$$

And the constant k is calculated from equation (2), where the time of reaction is the same in all the experiments.

From Arrhenius equation:

$$k = A\, e^{\frac{-Ea}{RT}} \qquad (3)$$

Transformed in linear form:

$$lnk = lnA - \frac{Ea}{R} \cdot \frac{1}{T} \qquad (4)$$

We will use the linear regression to calculate the Activation Energy (Ea) and the pre-exponential factor (A).

By the linear fit: ln k =f(1/T) we can calculate the slope, equal with Ea/R and, the intercept that will be equal with ln A.

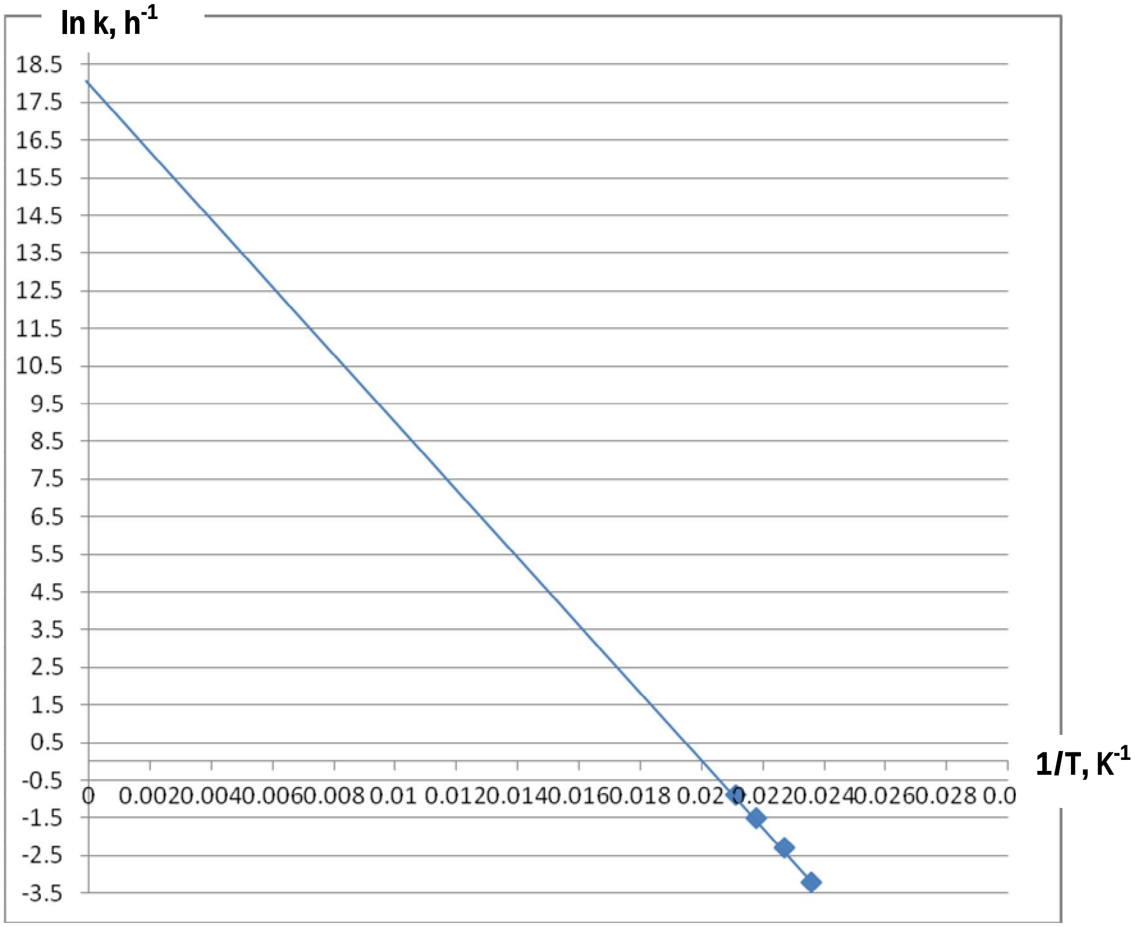

(the correlation factor R^2 = 0,9978)

The slope of the linear fit is: Ea/R = 8840.5

And Ea = 8840.5 x 8.314 = 73 499.9 = 73.5 kJ/mole

The intercept is ln A = 17.79

And, A = $e^{17.79}$ = $5.33 \times 10^7 h^{-1}$

TGA-DTA analysis data are presented in the following tables:

Sample 1 : Pure Terephthalic Acid

#FILE:	proba 0-AT Pure TPA
#FORMAT:	NETZSCH5
#FTYPE:	ANSI
#IDENTITY:	proba 0-AT refacut
#DECIMAL:	POINT
#SEPARATOR:	COMMA
#MTYPE:	TG
#INSTRUMENT:	NETZSCH TG 209F3
#PROJECT:	
#DATE/TIME:	7/2/2018 12:43:05 PM (UTC+2)
#CORR. FILE:	
#TEMPCAL:	TCALZERO.TCX
#LABORATORY:	CCM
#OPERATOR:	Aurel
#REMARK:	
#SAMPLE:	proba 0-AT refacut
#SAMPLE MASS /mg:	4.8755
#MATERIAL:	
#TYPE OF CRUCIBLE:	Al2O3
#SAMPLE CRUCIBLE MASS /mg:	0
#REFERENCE CRUCIBLE MASS /mg:	
#PROTECTIVE GAS:	NITROGEN
#FLOW RATE /(ml/min):	20
#PURGE GAS 1:	NITROGEN
#FLOW RATE 1 /(ml/min):	20
#PURGE GAS 2:	<no gas>
#FLOW RATE 2:	
#M.RANGE /mg:	2000
#CORR. CODE:	700
#RANGE:	25°C/10.0(K/min)/500°C
#SEGMENT:	S1/1
#SEG. 1:	25°C/10.0(K/min)/500°C

##Temp./°C	Time/min	Mass/%	DTG/(%/min)
25.737	0	100	-0.1205
28.237	0.99349	100.1525	0.25532
30.737	1.33055	100.2191	0.13378
33.237	1.61715	100.2519	9.78E-02

35.737	1.87947	100.2756	9.69E-02
38.237	2.12766	100.303	0.10639
40.737	2.36668	100.3209	4.00E-02
43.237	2.59917	100.3218	-1.92E-02
45.737	2.82753	100.3171	-2.61E-02
48.237	3.0536	100.3109	-1.88E-02
50.737	3.27779	100.3074	-2.24E-02
53.237	3.50127	100.3008	-3.41E-02
55.737	3.72472	100.292	-4.07E-02
58.237	3.94835	100.2874	-9.01E-03
60.737	4.17283	100.2795	-5.24E-02
63.237	4.39818	100.2688	-4.24E-02
65.737	4.6249	100.257	-6.94E-02
68.237	4.85253	100.2402	-7.26E-02
70.737	5.08184	100.2254	-6.22E-02
73.237	5.31275	100.2103	-5.27E-02
75.737	5.54528	100.2017	-3.66E-02
78.237	5.77898	100.1908	-4.79E-02
80.737	6.01396	100.1805	-4.84E-02
83.237	6.2506	100.1664	-7.02E-02
85.737	6.48837	100.1495	-6.56E-02
88.237	6.72729	100.135	-4.97E-02
90.737	6.96762	100.1247	-5.18E-02
93.237	7.2089	100.112	-3.64E-02
95.737	7.45052	100.0996	-9.58E-02
98.237	7.69322	100.0728	-8.21E-02
100.737	7.93547	100.0634	-1.90E-02
103.237	8.17848	100.0561	-4.84E-02
105.737	8.42209	100.0449	-3.63E-02
108.237	8.66578	100.0351	-5.51E-02
110.737	8.90991	100.0177	-8.46E-02
113.237	9.15415	99.99669	-7.48E-02
115.737	9.3983	99.97844	-8.08E-02
118.237	9.64244	99.96542	-3.36E-02
120.737	9.88694	99.95543	-4.49E-02
123.237	10.13115	99.94186	-7.25E-02
125.737	10.37494	99.9243	-6.24E-02
128.237	10.6189	99.91434	-1.75E-02
130.737	10.86282	99.90878	-4.30E-02
133.237	11.10684	99.89154	-8.61E-02
135.737	11.35053	99.87775	-3.14E-02
138.237	11.59427	99.86366	-7.93E-02
140.737	11.83842	99.84867	-3.63E-02

143.237	12.08294	99.84518	-9.30E-04
145.737	12.3276	99.83847	-6.33E-02
148.237	12.57263	99.82487	-3.28E-02
150.737	12.81787	99.81718	-4.10E-02
153.237	13.06356	99.803	-6.24E-02
155.737	13.30904	99.78975	-3.79E-02
158.237	13.555	99.78481	-1.88E-02
160.737	13.80154	99.77526	-5.68E-02
163.237	14.04836	99.75791	-7.71E-02
165.737	14.29543	99.74312	-4.03E-02
168.237	14.5429	99.73769	-1.52E-02
170.737	14.79025	99.73029	-4.03E-02
173.237	15.03812	99.72157	-2.20E-02
175.737	15.28592	99.71847	-1.92E-02
178.237	15.53403	99.69892	-0.13573
180.737	15.78223	99.6745	-3.25E-02
183.237	16.03074	99.67074	-3.09E-02
185.737	16.27941	99.65412	-8.29E-02
188.237	16.52842	99.64274	-1.27E-02
190.737	16.77736	99.63493	-5.94E-02
193.237	17.02528	99.6177	-5.98E-02
195.737	17.27384	99.61122	9.13E-04
198.237	17.52332	99.60902	-2.53E-02
200.737	17.77264	99.59144	-0.12633
203.237	18.02145	99.56821	-3.18E-02
205.737	18.2703	99.55818	-9.16E-02
208.237	18.51965	99.52716	-0.10744
210.737	18.76882	99.5035	-0.14412
213.237	19.01873	99.45719	-0.17142
215.737	19.26726	99.42575	-9.66E-02
218.237	19.51553	99.40443	-8.50E-02
220.737	19.7648	99.35557	-0.29852
223.237	20.01496	99.30302	-0.10461
225.737	20.26439	99.27227	-0.17704
228.237	20.51364	99.22159	-0.20419
230.737	20.7631	99.1733	-0.21158
233.237	21.0122	99.11065	-0.29879
235.737	21.26135	99.04369	-0.20599
238.237	21.51058	98.99111	-0.25431
240.737	21.75976	98.91405	-0.34012
243.237	22.00901	98.82455	-0.37578
245.737	22.25817	98.71816	-0.51796
248.237	22.5072	98.58388	-0.51178

250.737	22.75626	98.45581	-0.53295
253.237	23.00563	98.30335	-0.70017
255.737	23.25502	98.12362	-0.78604
258.237	23.50452	97.8893	-1.02024
260.737	23.75396	97.64165	-0.96354
263.237	24.0032	97.38375	-1.24397
265.737	24.25307	97.03086	-1.44588
268.237	24.50281	96.67748	-1.51262
270.737	24.7528	96.2389	-1.89671
273.237	25.00278	95.76344	-1.95558
275.737	25.25265	95.19437	-2.56082
278.237	25.50238	94.54039	-2.70807
280.737	25.75231	93.82615	-3.03884
283.237	26.00219	92.99944	-3.53665
285.737	26.25221	92.04917	-4.12562
288.237	26.50248	90.94168	-4.69344
290.737	26.75245	89.65858	-5.76369
293.237	27.00308	88.14331	-6.34817
295.737	27.25364	86.38821	-7.39886
298.237	27.50449	84.49573	-7.89067
300.737	27.75529	82.37085	-9.03031
303.237	28.00616	79.99242	-9.99566
305.737	28.25736	77.31046	-11.3834
308.237	28.50824	74.2874	-12.7752
310.737	28.75927	70.86985	-14.3698
313.237	29.01077	67.06544	-15.9968
315.737	29.26195	62.81224	-17.9183
318.237	29.51304	58.04808	-19.9735
320.737	29.76421	52.80122	-21.9212
323.237	30.01532	46.97386	-24.3949
325.737	30.26644	40.63483	-26.1823
328.237	30.51691	33.84189	-27.7887
330.737	30.76526	26.99225	-27.2063
333.237	31.01024	20.47815	-25.6159
335.737	31.25249	14.57921	-23.3342
338.237	31.49482	9.08967	-21.7596
340.737	31.74118	4.06333	-18.9836
343.237	31.98116	0.2064	-11.5364
345.737	32.21217	-1.13056	-1.45927
348.237	32.44562	-1.2226	-2.44E-02
350.737	32.6893	-1.23588	-0.10228
353.237	32.93956	-1.26405	-0.12528
355.737	33.19339	-1.29468	-0.13262

358.237	33.4491	-1.32702	-9.57E-02
360.737	33.70481	-1.34364	4.91E-04
363.237	33.96229	-1.31193	0.1217
365.737	34.21677	-1.33784	-0.21889
368.237	34.46832	-1.37072	-5.44E-02
370.737	34.72001	-1.3863	-5.82E-02
373.237	34.97215	-1.40546	-0.12374
375.737	35.22397	-1.42393	3.72E-03
378.237	35.47552	-1.42372	-6.50E-02
380.737	35.72747	-1.45443	-8.41E-02
383.237	35.97834	-1.46729	-0.10048
385.737	36.22927	-1.50435	-0.12572
388.237	36.47968	-1.51643	-2.04E-03
390.737	36.73011	-1.52006	-5.07E-02
393.237	36.98016	-1.54797	-0.16924
395.737	37.23047	-1.58145	-3.95E-02
398.237	37.4805	-1.5803	-8.42E-03
400.737	37.7304	-1.59463	-7.86E-02
403.237	37.98034	-1.61441	-6.66E-02
405.737	38.23001	-1.63247	-9.40E-02
408.237	38.47971	-1.6518	-7.56E-02
410.737	38.72982	-1.67609	-6.70E-02
413.237	38.97968	-1.68243	-3.39E-02
415.737	39.22988	-1.70591	-0.11567
418.237	39.47949	-1.72322	-4.28E-02
420.737	39.72924	-1.74506	-0.11411
423.237	39.97917	-1.76251	-2.39E-02
425.737	40.22928	-1.77092	-8.63E-02
428.237	40.47917	-1.80502	-0.1174
430.737	40.72891	-1.82631	-0.10056
433.237	40.97865	-1.84845	-3.37E-02
435.737	41.22836	-1.85126	-5.53E-02
438.237	41.47865	-1.881	-0.11032
440.737	41.72901	-1.9063	-0.12294
443.237	41.97941	-1.94008	-0.1201
445.737	42.22941	-1.96147	-6.06E-02
448.237	42.47982	-1.9727	-3.81E-02
450.737	42.72968	-1.98918	-0.10337
453.237	42.98004	-2.01868	-0.11777
455.737	43.23028	-2.04663	-9.67E-02
458.237	43.47981	-2.06546	-7.55E-02
460.737	43.72967	-2.09141	-0.10818
463.237	43.98012	-2.10934	-4.97E-02

465.737	44.22977	-2.12799	-9.86E-02
468.237	44.47952	-2.15255	-9.65E-02
470.737	44.72938	-2.17696	-0.10264
473.237	44.97931	-2.204	-9.84E-02
475.737	45.22859	-2.22423	-7.52E-02
478.237	45.47803	-2.24229	-0.10568
480.737	45.72751	-2.28361	-0.17289
483.237	45.97733	-2.30591	-1.05E-02
485.737	46.22675	-2.30692	-3.00E-02
488.237	46.47596	-2.31878	-2.52E-02
490.737	46.72527	-2.346	-0.2946
493.237	46.97462	-2.41059	-9.50E-02
495.737	47.22429	-2.4095	4.16E-02
498.237	47.47366	-2.41658	-9.52E-02

Sample 2 – Terephthalic Acid from Black Sea Experiment

#FILE:	proba 1 - AT.ngb-st6
#FORMAT:	NETZSCH5
#FTYPE:	ANSI
#IDENTITY:	proba 1 - AT
#DECIMAL:	POINT
#SEPARATOR:	COMMA
#MTYPE:	TG
#INSTRUMENT:	NETZSCH TG 209F3
#PROJECT:	
#DATE/TIME:	7/2/2018 11:02:19 AM (UTC+2)
#CORR. FILE:	
#TEMPCAL:	TCALZERO.TCX
#LABORATORY:	CCM
#OPERATOR:	Aurel
#REMARK:	
#SAMPLE:	proba 1 - AT
#SAMPLE MASS /mg:	3.234
#MATERIAL:	
#TYPE OF CRUCIBLE:	Al2O3
#SAMPLE CRUCIBLE MASS /mg:	0
#REFERENCE CRUCIBLE MASS /mg:	
#PROTECTIVE GAS:	NITROGEN
#FLOW RATE /(ml/min):	20
#PURGE GAS 1:	NITROGEN

#FLOW RATE 1 /(ml/min):		20		
#PURGE GAS 2:		<no gas>		
#FLOW RATE 2:				
#M.RANGE /mg:		2000		
#CORR. CODE:		700		
#RANGE:		25°C/10.0(K/min)/500°C		
#SEGMENT:		S1/1		
#SEG. 1:		25°C/10.0(K/min)/500°C		
##Temp./°C		Time/min	Mass/%	c-DTA®/K
	25.005	0	100	0.00E+00
	27.505	0.94053	100.0534	-6.89234
	30.005	1.26822	100.0829	-7.65497
	32.505	1.54918	100.0775	-7.95229
	35.005	1.80819	100.0465	-8.03158
	37.505	2.05404	99.99724	-7.97797
	40.005	2.2913	99.94427	-7.84177
	42.505	2.52328	99.88718	-7.65305
	45.005	2.75099	99.82504	-7.42695
	47.505	2.9764	99.75583	-7.17302
	50.005	3.20046	99.68827	-6.90433
	52.505	3.42392	99.61748	-6.63591
	55.005	3.64713	99.54866	-6.35716
	57.505	3.87097	99.48362	-6.08859
	60.005	4.09576	99.41423	-5.82723
	62.505	4.32161	99.33211	-5.58003
	65.005	4.54874	99.24729	-5.34503
	67.505	4.77715	99.15231	-5.11855
	70.005	5.00731	99.047	-4.91211
	72.505	5.23931	98.93497	-4.72774
	75.005	5.47225	98.81823	-4.54996
	77.505	5.7067	98.71245	-4.38583
	80.005	5.94234	98.60347	-4.23473
	82.505	6.17868	98.50516	-4.09297
	85.005	6.41681	98.41079	-3.96595
	87.505	6.65597	98.31843	-3.84643
	90.005	6.89603	98.226	-3.74165
	92.505	7.13696	98.13249	-3.64317
	95.005	7.37883	98.06164	-3.55216
	97.505	7.62129	97.992	-3.47253
	100.005	7.86395	97.91784	-3.38921
	102.505	8.10733	97.83011	-3.31472

105.005	8.35056	97.73869	-3.23943
107.505	8.59435	97.66083	-3.17283
110.005	8.83859	97.57995	-3.10321
112.505	9.0829	97.50848	-3.03919
115.005	9.32687	97.4353	-2.9725
117.505	9.57091	97.36938	-2.90324
120.005	9.81526	97.31153	-2.84243
122.505	10.05934	97.24113	-2.77141
125.005	10.30346	97.1614	-2.70751
127.505	10.54716	97.09243	-2.63676
130.005	10.79101	97.03108	-2.56708
132.505	11.03501	96.96935	-2.49906
135.005	11.27857	96.8938	-2.42644
137.505	11.52237	96.81065	-2.35551
140.005	11.7666	96.73211	-2.28948
142.505	12.01117	96.64269	-2.22963
145.005	12.25572	96.55562	-2.16728
147.505	12.50066	96.45905	-2.10548
150.005	12.74587	96.37376	-2.05159
152.505	12.99146	96.28194	-2.00093
155.005	13.23727	96.19754	-1.95048
157.505	13.48315	96.11237	-1.90027
160.005	13.72955	96.02912	-1.85845
162.505	13.97644	95.92807	-1.8185
165.005	14.22318	95.82995	-1.77675
167.505	14.47039	95.73016	-1.74033
170.005	14.71802	95.64729	-1.70986
172.505	14.96553	95.58146	-1.67726
175.005	15.2134	95.50619	-1.64658
177.505	15.46181	95.4179	-1.62253
180.005	15.71026	95.32412	-1.60239
182.505	15.95849	95.24201	-1.57353
185.005	16.20691	95.17528	-1.55242
187.505	16.45543	95.10896	-1.52833
190.005	16.7038	95.03211	-1.50178
192.505	16.95239	94.95062	-1.4823
195.005	17.20125	94.9038	-1.46355
197.505	17.45013	94.84493	-1.44323
200.005	17.69876	94.77767	-1.42036
202.505	17.94794	94.70954	-1.40546
205.005	18.19728	94.62551	-1.39191
207.505	18.44625	94.54039	-1.36953
210.005	18.69541	94.45339	-1.35595

212.505	18.94489	94.36884	-1.34201
215.005	19.19439	94.29863	-1.33091
217.505	19.44396	94.21371	-1.31708
220.005	19.69327	94.08949	-1.3014
222.505	19.9426	93.97367	-1.28358
225.005	20.19177	93.86184	-1.26927
227.505	20.44099	93.73207	-1.25411
230.005	20.69	93.58837	-1.23664
232.505	20.93886	93.43163	-1.21639
235.005	21.18841	93.26797	-1.20511
237.505	21.43772	93.09424	-1.19098
240.005	21.68659	92.90064	-1.17148
242.505	21.93564	92.67007	-1.15038
245.005	22.18487	92.38096	-1.13542
247.505	22.43421	92.10201	-1.12262
250.005	22.68342	91.78896	-1.10699
252.505	22.93254	91.43477	-1.0888
255.005	23.18186	91.04728	-1.07434
257.505	23.4311	90.62091	-1.06132
260.005	23.68075	90.14595	-1.04722
262.505	23.93046	89.60315	-1.03608
265.005	24.18031	88.99284	-1.02602
267.505	24.42997	88.32671	-1.01201
270.005	24.67956	87.59235	-1.00318
272.505	24.92951	86.75122	-0.99381
275.005	25.17928	85.81346	-0.98584
277.505	25.42889	84.74141	-0.97284
280.005	25.67904	83.53115	-0.96791
282.505	25.92917	82.17114	-0.95986
285.005	26.17907	80.65282	-0.94966
287.505	26.42928	78.91203	-0.94354
290.005	26.67945	76.81317	-0.93745
292.505	26.92957	74.49645	-0.9304
295.005	27.18002	71.85728	-0.92331
297.505	27.43032	69.00256	-0.92417
300.005	27.68072	65.8712	-0.91639
302.505	27.93107	62.37015	-0.91471
305.005	28.18189	58.47024	-0.91401
307.505	28.43241	54.19021	-0.91097
310.005	28.68301	49.51603	-0.90961
312.505	28.93364	44.37764	-0.90905
315.005	29.18432	38.83217	-0.90258
317.505	29.43424	32.92965	-0.89768

320.005	29.6845	26.63569	-0.89141
322.505	29.93394	20.16282	-0.88093
325.005	30.18121	13.92598	-0.84358
327.505	30.42645	8.3224	-0.78683
330.005	30.66936	3.98412	-0.71189
332.505	30.906	2.23128	-0.56578
335.005	31.14345	1.94942	-0.43366
337.505	31.38764	1.80605	-0.36918
340.005	31.63644	1.69858	-0.34939
342.505	31.88787	1.60256	-0.35379
345.005	32.14044	1.51086	-0.37261
347.505	32.3932	1.4326	-0.39141
350.005	32.64598	1.34817	-0.41116
352.505	32.89846	1.27059	-0.42932
355.005	33.15042	1.19224	-0.43873
357.505	33.40216	1.11053	-0.44836
360.005	33.6536	1.0194	-0.45361
362.505	33.90426	0.91055	-0.45424
365.005	34.15499	0.76778	-0.45131
367.505	34.40567	0.66688	-0.45321
370.005	34.65621	0.55824	-0.44852
372.505	34.90628	0.46153	-0.44095
375.005	35.15633	0.36071	-0.43225
377.505	35.40616	0.25425	-0.42377
380.005	35.65621	0.1443	-0.41782
382.505	35.90619	1.29E-02	-0.40814
385.005	36.15604	-0.11017	-0.39793
387.505	36.4059	-0.24678	-0.39056
390.005	36.65537	-0.37059	-0.37664
392.505	36.9053	-0.47319	-0.36473
395.005	37.1553	-0.58352	-0.35844
397.505	37.40491	-0.68467	-0.34552
400.005	37.65521	-0.7796	-0.34237
402.505	37.90534	-0.85796	-0.33639
405.005	38.15478	-0.93498	-0.32136
407.505	38.4043	-1.01873	-0.30917
410.005	38.65412	-1.10533	-0.29938
412.505	38.90409	-1.17925	-0.29099
415.005	39.15356	-1.25867	-0.27493
417.505	39.40344	-1.36242	-0.26793
420.005	39.65298	-1.45056	-0.25524
422.505	39.90297	-1.52125	-0.24611
425.005	40.15275	-1.60515	-0.23753

427.505	40.40298	-1.68195	-0.23119
430.005	40.65322	-1.76587	-0.22621
432.505	40.90308	-1.82455	-0.21776
435.005	41.15289	-1.87023	-0.20599
437.505	41.40301	-1.93523	-0.20013
440.005	41.65328	-1.99237	-0.19342
442.505	41.90343	-2.04439	-0.18537
445.005	42.1537	-2.09562	-0.18216
447.505	42.40407	-2.16173	-0.17731
450.005	42.65417	-2.21419	-0.16837
452.505	42.90477	-2.29251	-0.17013
455.005	43.15501	-2.3586	-0.16202
457.505	43.40548	-2.425	-0.15975
460.005	43.65541	-2.47965	-0.15098
462.505	43.90498	-2.53166	-0.13985
465.005	44.15512	-2.58624	-0.12976
467.505	44.40473	-2.65267	-0.11581
470.005	44.65434	-2.72165	-0.10851
472.505	44.90363	-2.78143	-8.99E-02
475.005	45.1533	-2.84225	-7.85E-02
477.505	45.4031	-2.91916	-6.85E-02
480.005	45.65238	-2.99259	-5.38E-02
482.505	45.90229	-3.0667	-4.72E-02
485.005	46.15186	-3.13831	-3.36E-02
487.505	46.40133	-3.20329	-2.29E-02
490.005	46.65124	-3.2697	-1.33E-02
492.505	46.90092	-3.32869	-1.66E-03
495.005	47.15062	-3.3819	1.01E-02
497.505	47.40064	-3.44125	2.20E-02

Sample 3 – Terephthalic Acid from Atlantic Ocean experiment

#FILE:	proba 2-AT.ngb-st6
#FORMAT:	NETZSCH5
#FTYPE:	ANSI

#IDENTITY:	proba 2-AT
#DECIMAL:	POINT
#SEPARATOR:	COMMA
#MTYPE:	TG
#INSTRUMENT:	NETZSCH TG 209F3
#PROJECT:	
#DATE/TIME:	7/2/2018 2:35:38 PM (UTC+2)
#CORR. FILE:	
#TEMPCAL:	TCALZERO.TCX
#LABORATORY:	CCM
#OPERATOR:	Aurel
#REMARK:	
#SAMPLE:	proba 2-AT
#SAMPLE MASS /mg:	6.0139
#MATERIAL:	
#TYPE OF CRUCIBLE:	Al2O3
#SAMPLE CRUCIBLE MASS /mg:	0
#REFERENCE CRUCIBLE MASS /mg:	
#PROTECTIVE GAS:	NITROGEN
#FLOW RATE /(ml/min):	20
#PURGE GAS 1:	NITROGEN
#FLOW RATE 1 /(ml/min):	20
#PURGE GAS 2:	<no gas>
#FLOW RATE 2:	
#M.RANGE /mg:	2000
#CORR. CODE:	700
#RANGE:	25°C/10.0(K/min)/500°C
#SEGMENT:	S1/1
#SEG. 1:	25°C/10.0(K/min)/500°C

##Temp./°C	Time/min	Mass/%	c-DTA®/K
25.096	0	100	0.00E+00
27.596	0.9538	100.1523	-7.02089
30.096	1.28262	100.2102	-7.78845
32.596	1.56388	100.248	-8.09121
35.096	1.82317	100.2732	-8.17362
37.596	2.06886	100.29	-8.12391
40.096	2.30576	100.3001	-7.98108
42.596	2.53775	100.3033	-7.79301
45.096	2.76551	100.3101	-7.5619
47.596	2.99084	100.3107	-7.30718

50.096	3.21317	100.311	-7.02495
52.596	3.43595	100.3083	-6.74049
55.096	3.65943	100.3071	-6.47304
57.596	3.88323	100.3023	-6.19928
60.096	4.10775	100.2994	-5.93429
62.596	4.33345	100.2929	-5.68856
65.096	4.56007	100.2858	-5.4452
67.596	4.78843	100.2795	-5.22078
70.096	5.01793	100.2665	-5.00702
72.596	5.24912	100.2557	-4.81002
75.096	5.4815	100.2478	-4.62642
77.596	5.71485	100.2334	-4.4544
80.096	5.95059	100.2267	-4.30398
82.596	6.18756	100.2171	-4.16446
85.096	6.42526	100.2062	-4.03267
87.596	6.66424	100.1893	-3.91549
90.096	6.90413	100.1774	-3.80416
92.596	7.14511	100.1651	-3.70576
95.096	7.38683	100.1544	-3.61573
97.596	7.62948	100.1411	-3.53144
100.096	7.87203	100.1307	-3.45087
102.596	8.11517	100.1205	-3.37314
105.096	8.35908	100.1043	-3.3028
107.596	8.60286	100.092	-3.23265
110.096	8.84725	100.079	-3.17007
112.596	9.09109	100.0639	-3.09623
115.096	9.33504	100.0544	-3.03006
117.596	9.57961	100.038	-2.97061
120.096	9.82398	100.0249	-2.9026
122.596	10.06758	100.0104	-2.82687
125.096	10.31151	99.99357	-2.76132
127.596	10.55562	99.98181	-2.69278
130.096	10.79974	99.96468	-2.62355
132.596	11.04321	99.94011	-2.55306
135.096	11.28728	99.91313	-2.48551
137.596	11.5312	99.89266	-2.41545
140.096	11.77585	99.86613	-2.35318
142.596	12.01998	99.85037	-2.2872
145.096	12.26424	99.8347	-2.21908
147.596	12.50892	99.80186	-2.15881
150.096	12.75409	99.77893	-2.10297
152.596	12.99999	99.75554	-2.0503
155.096	13.24575	99.7239	-1.99881

157.596	13.49205	99.69103	-1.95532
160.096	13.73884	99.66429	-1.91513
162.596	13.98557	99.64038	-1.87399
165.096	14.23248	99.61143	-1.83468
167.596	14.47952	99.57987	-1.79709
170.096	14.72744	99.54498	-1.76736
172.596	14.97509	99.51015	-1.73298
175.096	15.22305	99.46726	-1.70347
177.596	15.47087	99.41074	-1.67438
180.096	15.7189	99.35459	-1.6478
182.596	15.96676	99.28834	-1.61815
185.096	16.21527	99.23798	-1.59315
187.596	16.46379	99.22156	-1.5697
190.096	16.71244	99.16571	-1.54624
192.596	16.96143	99.09698	-1.52894
195.096	17.21042	99.02881	-1.50956
197.596	17.4594	98.952	-1.49201
200.096	17.70857	98.88728	-1.47513
202.596	17.95798	98.81762	-1.4594
205.096	18.20706	98.75024	-1.44203
207.596	18.45609	98.67649	-1.42446
210.096	18.70499	98.5944	-1.40596
212.596	18.95463	98.52681	-1.39431
215.096	19.20396	98.45819	-1.37594
217.596	19.45321	98.38141	-1.36142
220.096	19.70279	98.3158	-1.34732
222.596	19.95198	98.23248	-1.33179
225.096	20.20139	98.14508	-1.31668
227.596	20.45059	98.066	-1.30049
230.096	20.69985	97.97391	-1.28504
232.596	20.94924	97.88052	-1.26943
235.096	21.19856	97.77721	-1.25567
237.596	21.44804	97.67226	-1.24193
240.096	21.69686	97.59363	-1.21976
242.596	21.94563	97.48116	-1.19966
245.096	22.19463	97.36442	-1.18132
247.596	22.44397	97.24603	-1.16803
250.096	22.69343	97.10311	-1.15131
252.596	22.94269	96.96156	-1.13406
255.096	23.19207	96.80873	-1.12056
257.596	23.44154	96.61565	-1.10732
260.096	23.69105	96.40796	-1.09279
262.596	23.94052	96.20296	-1.07947

265.096	24.19006	95.95052	-1.06525
267.596	24.43974	95.65167	-1.05471
270.096	24.68911	95.3186	-1.03925
272.596	24.93891	94.93142	-1.03166
275.096	25.18879	94.50239	-1.01974
277.596	25.4387	93.99978	-1.00878
280.096	25.68831	93.43378	-0.99787
282.596	25.93815	92.78598	-0.98691
285.096	26.18796	92.05951	-0.97787
287.596	26.4378	91.23564	-0.96703
290.096	26.6878	90.2812	-0.95828
292.596	26.93805	89.1235	-0.95138
295.096	27.18819	87.83162	-0.9443
297.596	27.43901	86.37001	-0.94554
300.096	27.68958	84.77391	-0.94119
302.596	27.9402	83.0165	-0.9365
305.096	28.19086	81.0412	-0.9355
307.596	28.44151	78.82067	-0.93336
310.096	28.6922	76.32368	-0.92926
312.596	28.94323	73.5445	-0.93392
315.096	29.19377	70.48	-0.9309
317.596	29.4446	67.08954	-0.92964
320.096	29.69548	63.35939	-0.93294
322.596	29.94607	59.28514	-0.92624
325.096	30.19643	54.85995	-0.92201
327.596	30.44694	50.01028	-0.91752
330.096	30.6976	44.82313	-0.91554
332.596	30.94763	39.32754	-0.90852
335.096	31.19727	33.52647	-0.8972
337.596	31.44635	27.53651	-0.87521
340.096	31.69424	21.61435	-0.84868
342.596	31.93855	16.18074	-0.78312
345.096	32.17996	11.58384	-0.68985
347.596	32.41402	8.7039	-0.52266
350.096	32.6427	7.6705	-0.2965
352.596	32.87791	7.31893	-0.14126
355.096	33.12244	7.15382	-7.76E-02
357.596	33.37353	7.05434	-8.08E-02
360.096	33.62781	6.97466	-0.11544
362.596	33.88365	6.89278	-0.16815
365.096	34.13967	6.81927	-0.21507

367.596	34.3952	6.74809	-0.26424
370.096	34.65035	6.65618	-0.30737
372.596	34.90433	6.59263	-0.33861
375.096	35.15749	6.53293	-0.36236
377.596	35.4104	6.44854	-0.3811
380.096	35.66261	6.3683	-0.39617
382.596	35.91396	6.29764	-0.40175
385.096	36.16469	6.23235	-0.39715
387.596	36.41571	6.15538	-0.4015
390.096	36.66627	6.03755	-0.39725
392.596	36.9166	5.92916	-0.39147
395.096	37.16672	5.82129	-0.38326
397.596	37.41656	5.69614	-0.37431
400.096	37.66634	5.57424	-0.36161
402.596	37.91594	5.43519	-0.34916
405.096	38.16558	5.2854	-0.33601
407.596	38.41478	5.14527	-0.32319
410.096	38.66427	4.96935	-0.30754
412.596	38.91426	4.78703	-0.29997
415.096	39.16424	4.60971	-0.28954
417.596	39.41415	4.42443	-0.28142
420.096	39.66359	4.24615	-0.26606
422.596	39.91293	4.07095	-0.25168
425.096	40.16237	3.91446	-0.23629
427.596	40.41244	3.72983	-0.22983
430.096	40.66252	3.5919	-0.22001
432.596	40.91224	3.47539	-0.20963
435.096	41.16202	3.36308	-0.19643
437.596	41.41169	3.28935	-0.1836
440.096	41.66147	3.22467	-0.17639
442.596	41.91108	3.15481	-0.16246
445.096	42.16073	3.11488	-0.14907
447.596	42.41122	3.05293	-0.1456
450.096	42.66141	3.03675	-0.14
452.596	42.91155	3.00116	-0.13165
455.096	43.16214	2.94502	-0.12918
457.596	43.41316	2.90515	-0.13235
460.096	43.66368	2.87811	-0.12788
462.596	43.91417	2.85452	-0.12482
465.096	44.16447	2.83863	-0.11827
467.596	44.41432	2.79904	-0.10866
470.096	44.66435	2.7799	-0.10289
472.596	44.91471	2.74773	-9.64E-

			02
475.096	45.1645	2.70424	-8.27E-02
477.596	45.41411	2.68303	-7.12E-02
480.096	45.66384	2.66383	-6.28E-02
482.596	45.91365	2.64488	-5.16E-02
485.096	46.16345	2.61556	-3.93E-02
487.596	46.41296	2.59667	-2.57E-02
490.096	46.66275	2.57037	-1.58E-02
492.596	46.91246	2.54006	-2.31E-03
495.096	47.16217	2.50958	6.37E-03
497.596	47.41194	2.49878	1.91E-02
